# MiR-146a-5p targeting *SMAD4* and *TRAF6* inhibits adipogenensis through TGF-β and AKT/mTORC1 signal pathways in porcine intramuscular preadipocytes

**DOI:** 10.1186/s40104-020-00525-3

**Published:** 2021-02-03

**Authors:** Que Zhang, Rui Cai, Guorong Tang, Wanrong Zhang, Weijun Pang

**Affiliations:** Laboratory of Animal Fat Deposition and Muscle Development, College of Animal Science and Technology, Northwest A&F University, Yangling, 712100 Shaanxi China

**Keywords:** Adipogenesis, AKT/mTORC1 signal pathway, MiR-146a-5p, Porcine intramuscular fat, SMAD4, TGF-β signal pathway, TRAF6

## Abstract

**Background:**

Intramuscular fat (IMF) content is a vital parameter for assessing pork quality. Increasing evidence has shown that microRNAs (miRNAs) play an important role in regulating porcine IMF deposition. Here, a novel miRNA implicated in porcine IMF adipogenesis was found, and its effect and regulatory mechanism were further explored with respect to intramuscular preadipocyte proliferation and differentiation.

**Results:**

By porcine adipose tissue miRNA sequencing analysis, we found that miR-146a-5p is a potential regulator of porcine IMF adipogenesis. Further studies showed that miR-146a-5p mimics inhibited porcine intramuscular preadipocyte proliferation and differentiation, while the miR-146a-5p inhibitor promoted cell proliferation and adipogenic differentiation. Mechanistically, miR-146a-5p suppressed cell proliferation by directly targeting SMAD family member 4 (*SMAD4*) to attenuate TGF-β signaling. Moreover, miR-146a-5p inhibited the differentiation of intramuscular preadipocytes by targeting TNF receptor-associated factor 6 (*TRAF6*) to weaken the AKT/mTORC1 signaling downstream of the *TRAF6* pathway.

**Conclusions:**

MiR-146a-5p targets *SMAD4* and *TRAF6* to inhibit porcine intramuscular adipogenesis by attenuating TGF-β and AKT/mTORC1 signaling, respectively. These findings provide a novel miRNA biomarker for regulating intramuscular adipogenesis to promote pork quality.

**Supplementary Information:**

The online version contains supplementary material available at 10.1186/s40104-020-00525-3.

## Background

Intramuscular fat (IMF) content is implicated in pork tenderness, flavor, and juiciness, and is an important indicator for assessing pork quality [1, 2]. Therefore, proper ways to increase IMF content and improve pork quality has become an important topic in recent years [3]. The deposition of IMF is achieved through the proliferation and differentiation of intramuscular preadipocytes [4]. The proliferation of preadipocytes is regulated by differential expression of cell cycle regulators, including cyclin-dependent kinases (*CDKs*), CDK inhibitors (*CKIs*), and other transcription factors [5]. Similarly, the preadipocyte differentiation process also involves many regulatory factors, including the peroxisome proliferator-activated receptor *γ (PPARγ*), CCAAT/enhancer binding protein (*C/EBP*) family, fatty acid binding protein 4 (*FABP4*), and lipoprotein lipase (*LPL*) [6]. However, besides the above key genes, many miRNAs identified by RNA-seq are also involved in the regulation of porcine IMF content, and their functions and mechanisms require further study.

miRNAs are small, evolutionarily conserved non-coding RNAs that have an important function in regulating gene expression. miRNAs function generally by binding target genes that match their “seed sequences” to suppress or degrade target gene mRNAs after transcription and coordinate normal processes, including cellular proliferation, differentiation and apoptosis [7, 8]. Increasing evidence has shown that miRNAs, including miR-206 [9], miR-149-5p [10] and miR-204-5p [11], play key roles in preadipocyte proliferation and differentiation. Moreover, many miRNAs are differentially expressed in porcine adipose tissue during different developmental stages and have been studied using transcriptome sequencing technology [12]. We therefore speculated that these miRNAs may be key regulators of porcine IMF deposition through their target genes.

Classical TGF-β/SMAD signaling regulates cell proliferation, differentiation, migration and growth. SMAD4 is an important transmission medium that transduces extracellular signals, including TGF-β and BMP, to the nucleus [13–15]. It has been reported in the literature that SMAD4 regulates the proliferation and migration of A549 cells [16], dihydromyricetin inhibits the proliferation of human choriocarcinoma JAR cells via downregulation of *SMAD4* expression [17], and miR-224 mediates the proliferation of HCT116 cells by targeting *SMAD4* [18]. Although *SMAD4* has been discovered and studied in the proliferation of different cell types, it needs to be further explored whether *SMAD4* is implicated in proliferation of porcine intramuscular preadipocytes.

TRAF6 is an adapter protein that possesses E3 ubiquitin ligase activity [19] and plays an important role in intracellular signal transduction [20]. For instance, TRAF6 is a direct ubiquitinase of the serine/threonine kinase AKT and promotes AKT phosphorylation and activation [21, 22]. AKT is a node at the junction of many major intracellular signaling pathways [23] and plays an essential role in adipocyte differentiation. Preadipocytes that lack Akt exhibit differentiation defects because they fail to induce PPARγ expression at the beginning of the adipogenesis program [24–26]. Furthermore, mTORC1, a major downstream signaling regulator of AKT, is a key regulator of mRNA translation and cell growth [27]. Several studies have found that mTORC1 activity is required for proper preadipocyte differentiation [28–30]. However, whether TRAF6 acts as an miRNA target to regulate the activation of the AKT/mTORC1 signaling pathway, thereby affecting porcine intramuscular preadipocyte differentiation, remains unknown.

In this study, based on a published miRNA sequencing analysis of adipose and muscle tissue at four developmental stages of pigs, at one, 30, 90 and 240 days of age [31], we found that miR-146a-5p showed significant expression differences in adipose tissue at all stages, but was not differentially expressed in muscle tissue at a later stage of growth. These findings implying that miR-146a-5p may have a key effect on IMF deposition, and our results reveal that miR-146a-5p inhibits intramuscular preadipocyte proliferation and differentiation by targeting *SMAD4* and *TRAF6* through TGF-β and AKT/mTORC1 signaling pathways. Thus, miR-146a-5p is a novel key regulator of pig IMF deposition.

## Methods

### Animal and sample collection

Piglets (3 days old) were provided by the animal experiment animal ranch of Northwest A&F University. According to the regulations of the Animal Protection Committee of Northwest A&F University, all pigs were killed in the slaughterhouse. Dissect the heart, liver, spleen, lung, kidney, *longissimus dorsi* muscle (LD) and subcutaneous white adipose tissue (SWAT), and rinse with phosphate buffered saline (PBS). The samples used for real-time quantitative PCR (RT-qPCR) were frozen and stored in liquid nitrogen.

### Isolation and culture of porcine intramuscular preadipocytes

At 3 days old piglets were sacrificed, the intramuscular preadipocytes in LD were extracted as previously described [32]. Cells were re-suspended in DMEM/F12 and plated at a density 6 × 10^5^ per 60-mm culture dish (Fig. S1A), and cultured in a 5% CO_2_ incubator at 37 °C. When the cells grow to confluence (Fig. S1C), the medium was changed with adipogenic induction medium, which is the DMEM/F12 supplement with 10% FBS, 100 U/mL penicillin-streptomycin, 0.5 mmol/L IBMX, 1 nmol/L DEX, and 5 ng/mL insulin (IBMX, DEX and insulin were purchased from Sigma).

### Transfection of mimics/inhibitor NC and miR-146a-5p mimic/inhibitor

Porcine intramuscular preadipocytes were seeded in 6-well, 12-well, 24-well or 96-well plates. When detecting cell proliferation, miR-146a-5p mimics or mimics negative control (MNC) (Ribobio, China) were transfected (50 nmol/L) when the cell density reached 50–60% (Fig. S1B). During transfection, X-tremeGENE siRNA Transfection Reagent (Roche, USA) was mixed with Opti-MEM medium (Gibco, USA) for 5 min, then the two mixtures were mixed for 20 min and added to the cell culture medium, and the medium was replaced with fresh culture after 12 h. Cells were harvested 24 h after transfection for cell proliferation studies. When transfected with miR-146a-5p inhibitor, the method is the same as above, but the final concentration of miR-146a-5p inhibitor was 100 nmol/L. For the differentiation of preadipocytes, the cells were transfected when the cell density reached 70%. When cells reached confluence after transfection, adipogenic differentiation was induced by switching to differentiation medium.

### Total RNA extraction, RNA reverse transcription and RT-qPCR

After obtaining the cells, the cells were lysed with Trizol reagent (TakaRa, Otsu, Japan) and the total RNA in the cells was extracted. The concentration of total RNA was measured by the NanoDrop 2000 (Thermo, Waltham, MA, USA). Then the reverse transcription kit (TakaRa, Otsu, Japan) was used to synthesize cDNA. The specific reverse transcription primers and procedures were used for miRNA inversion. About real-time quantitative PCR, the SYBR green kit was used and three replicates were set up, and then the PCR reaction was performed on the Bio-Rad iQTM5 system. GAPDH was used as the internal reference for all genes for standardized analysis. But when analyzing miR-146a-5p levels, U6 was used as an internal reference. Table 1 shows the primer sequences used for qPCR. The primer sequences used for qPCR were shown in Table 1.

**Table 1:** Primer sequences used in this study

Gene	Accession number	Forward sequences (5′→3′)	Reverse sequences (5′→3′)
*Cyclin B*	NM_001285465.1	GCATCTTTGCTGAGATGGTGAC	AATCTTGCCTGGCCCACTTA
*Cyclin D*	NM_001123097.1	GGCCCTCAAGAGCGTAAGAG	GTCTCTCGATCAGTTCGGGC
*Cyclin E*	NM_001044621.3	GCCAGACTATAAGCCCTACCC	GGACCGGGTTACACTTCAGG
*P21*	XM_013977858.2	ACGTCTCAGGAGGACCATGT	AGAAGATCAGCCGGCGTTTG
*C/EBPβ*	NM_001199889.1	TCCGATCTCTTCTCCGACGA	CAGGCTCACGTAGCCGTATT
*PPARγ*	NM_001354666.3	AGGACTACCAAAGTGCCATCAAA	GAGGCTTTATCCCCACAGACAC
*FABP4*	NM_001002817.1	TGAAAGAAGTGGGAGTGGGC	CTGGCCCAATTTGAAGGCAA
*SMAD4*	NM_008540.3	TCACTATGAGCGGGTTGTCTC	TCCTTCAGTGGGTAAGGACG
*TRAF6*	NM_001105286.1	GGGAACGATACGCCTTACAA	CTCTGTCTTAGGGCGTCCAG
*GAPDH*	KJ786424	AGGTCGGAGTGAACGGATTTG	AGGTCGGAGTGAACGGATTTG

### Western blots

Cell samples were lysed using radio immunoprecipitation assay (RIPA) buffer (Beyotime, China) supplemented with protease inhibitor (Pierce, WA, USA) and total protein was extracted. The total protein samples were separated by electrophoresis in SDS-polyacrylamide gel. Then transferred it to PVDF membranes (Millipore, Bedford, MA, USA). After blocking the membrane in 5% skim milk for 2 h, the primary antibody was incubated overnight (4 °C) and the secondary antibody was incubated for 1.5 h (4 °C). Protein bands were exposed with chemiluminescent reagents (Millipore, Bedford, MA, USA) and quantified using Image J. Following primary antibodies were used: Cyclin D (1:100; Santa Cruz, USA), Cyclin E (1:100; Santa Cruz, USA), PCNA (1:1000; CST, USA), P21 (1:100; Santa Cruz, USA), C/EBPβ (1:100; Santa Cruz, USA), PPARγ (1:100; Santa Cruz, USA), FABP4 (1:100; Santa Cruz Biotechnology, Dallas, TX, USA), TRAF6 (1:500; Aviva Systems Biology, USA), SMAD4 (1:100; Santa Cruz, USA), β-actin (1:1000; Santa Cruz Biotechnology, Dallas, TX, USA), AKT (1:2000; Cell Signaling Technology, USA), p-AKT (1:2000; Cell Signaling Technology, USA), mTORC1 (1:2000; Cell Signaling Technology, USA), p-mTORC1 (1:2000; Cell Signaling Technology, USA),The secondary antibodies were anti-rabbit, anti-goat and anti-mouse antibodies (1:3000; Santa Cruz Biotechnology, Dallas, TX, USA). The targeted proteins were detected using the Gel Doc XR System (Bio-Rad, Hercules, CA, USA) as the instructions of the manufacturer.

### Target prediction and luciferase activity assay

The target genes of miR-146a-5p were predicted with Target-Scan 7.0. For the dual-reporter assay, we constructed a wild-type and mutant psiCHECK-2-reporter vector containing the target genes *SMAD4* and *TRAF6* 3′ UTR region (TongYong, Anhui, China). HEK293T was seeded in a 48-well plate and cotransfected with miRNA mimics or the negative control with psiCHECK-2-SMAD4 (or TRAF6)-reporter vector or mutant vector. After 48 h of transfection, the relative luciferase activity of Renilla compared with firefly was measured.

### EDU imaging assay

We used the Cell-Light™ EdU Apollo® 567 In Vitro Imaging Kit and configured the mixed solution according to the instructions. The preadipocytes in the normal growth stage were treated with 50 μmol/L EDU medium for 2 h. After the cells were fixed with 4% paraformaldehyde, they were stained with Apollo reaction solution. Then cell nucleus was stained with Hoechst. Nikon TE2000 microscope (Nikon, Tokyo, Japan) was used to take pictures, and the data was analyzed using Image J.

### Cell counting kit 8 (CCK8) analysis

Preadipocytes were seeded to 96-well plate in a number of 4 × 10^3^ cells. Preadipocytes were transfected with miR-146a-5p mimics/inhibitor or mimics/inhibitor negative control with 3 repetitions. After treatment for 24 h we switched the cells to culture medium containing 10% CCK solution for 2 h at 37 °C followed by measuring absorbance at 490 nm.

### Flow cytometry

Preadipocytes were seeded in 6- well culture plate at a density of 4 × 10^5^ cells per well. Cells were transfected with miR-146a-5p mimic or inhibitor for 24 h. After washed three times with PBS, cells were fixed with 70% alcohol overnight at − 20 °C followed by being treated with 1 mg/mL RNAase at 37 °C for 40 min, and stained with 50 mg/mL propidium iodide (PI) at 4 °C for 1 h. The samples were detected using a FACS Calibur flow cytometer (Becton Dickinson, Franklin Lakes, NJ, USA). The proliferation index (PI) shows the proportion of mitotic cells among the 10,000 cells examined.

### Oil Red O, BODIPY and AdipoRed staining

For Oil Red O staining, cells were fixed in a 4% paraformaldehyde solution for 30 min, induced with 60% Oil Red O for 30 min, and washed three times with PBS, and then the cells were visualized by phase-contrast microscope (IS-Elements software, Nikon ECLIPSE, Tokyo, Japan). Oil Red O was extracted with 100% isopropanol, and its relative concentration was determined by measuring the absorbance at 510 nm. After being fixed in 4% paraformaldehyde solution for 15 min, cells were stained with BODIPY (1 μg/mL; Life Technologies, Carlsbad, CA, USA) or AdipoRed (30 μL/mL; Lonza, USA) for 20 min; the sections were washed with PBS three times for 5 min each. For nuclear visualization, DAPI (4′,6-diamidino-2-phenylindole; Roche) was incubated for 10 min, then the section was rinsed with PBS. After treatment, the sections were observed under fluorescence microscope (Nikon, Tokyo, Japan).

### Bioinformatics analysis

The sequences of miRNAs were searched for at miRBase (http://www.mirbase.org/). Sequence alignment using MAGA software. The 3′ UTR sequences of E2F3 and P55PIK were downloaded from NCBI. Target genes of miRNA were predicted by TargetScan 7.0 Human (http://www.targetscan.org).

### Statistical analysis

All charts were created using GraphPad Prism 6.0 and the data represent the mean ± SEM. The significance of differences between the groups was assessed using the Student’s *t* test or one-way analysis (*, *P*<0.05; **, *P*<0.01).

## Results

### MiR-146a-5p is a potential regulator of porcine IMF adipogenesis

**Fig. 1:**
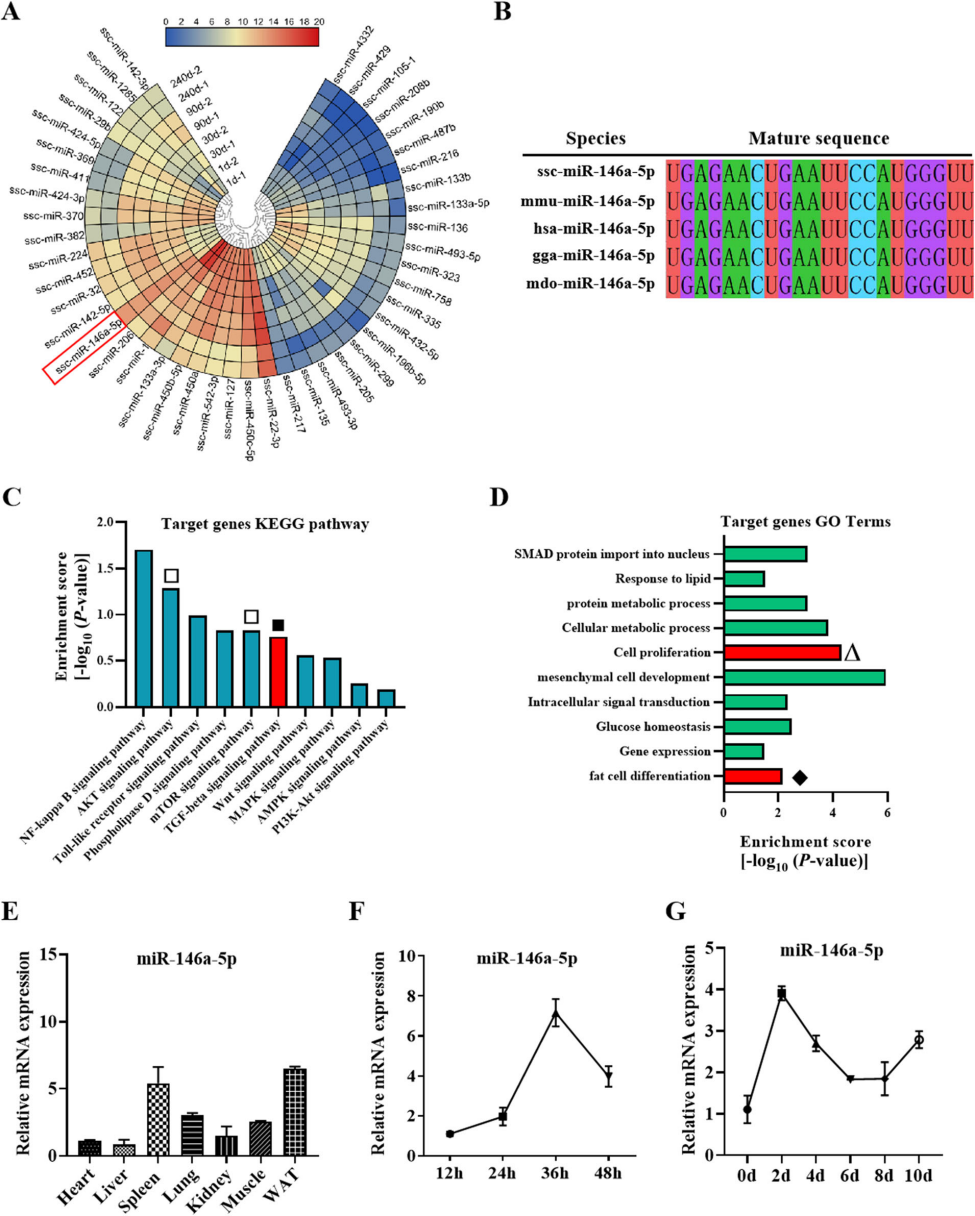
MiR-146a-5p is a potential regulator of porcine IMF adipogenesis. **a** The differential expression analysis heat map of miRNA sequencing data. Different colors represented the relative expression. **b** Comparation of miR-146a-5p seed sequence from pig, mice and human, etc. **c** KEGG pathway analysis of the miR-146a-5p target genes. **d** GO term analysis of the miR-146a-5p target genes. **e** Tissue expression profile of miR-146a-5p in pig. **f** RT-qPCR was performed to detect the expression of miR-146a-5p in proliferating in porcine intramuscular preadipocytes. **g** RT-qPCR analysis of miR-146a-5p expression after inducing adipogenic differentiation. Results are representative of the mean ± SEM (*n* = 3)

To identify miRNAs related to porcine IMF deposition, miRNA sequencing data were analyzed. As shown in the heat map, the levels of miR-146a-5p in the 30 d, 90 d, and 240 d porcine adipose tissue were significantly higher than those in the 0 d piglets (Fig.1a). Moreover, the miR-146a-5p seed sequence in humans, pigs and mice is highly conserved (Fig. 1b). The KEGG pathway analysis predicted that miR-146a-5p is involved in the TGF-β, AKT and mTORC pathways (Fig. 1c), and the GO term analysis suggested that miR-146a-5p can regulate cell proliferation and fat cell differentiation (Fig. 1d). Furthermore, miR-146a-5p is highly expressed in porcine WAT (Fig. 1e). Most importantly, the levels of miR-146a-5p increased first and then decreased in proliferated and differentiated porcine intramuscular preadipocytes, but they showed an upward trend in the late stage of differentiation (Fig. 1f and g).

### MiR-146a-5p mimics inhibit proliferation of porcine intramuscular preadipocytes

**Fig. 2:**
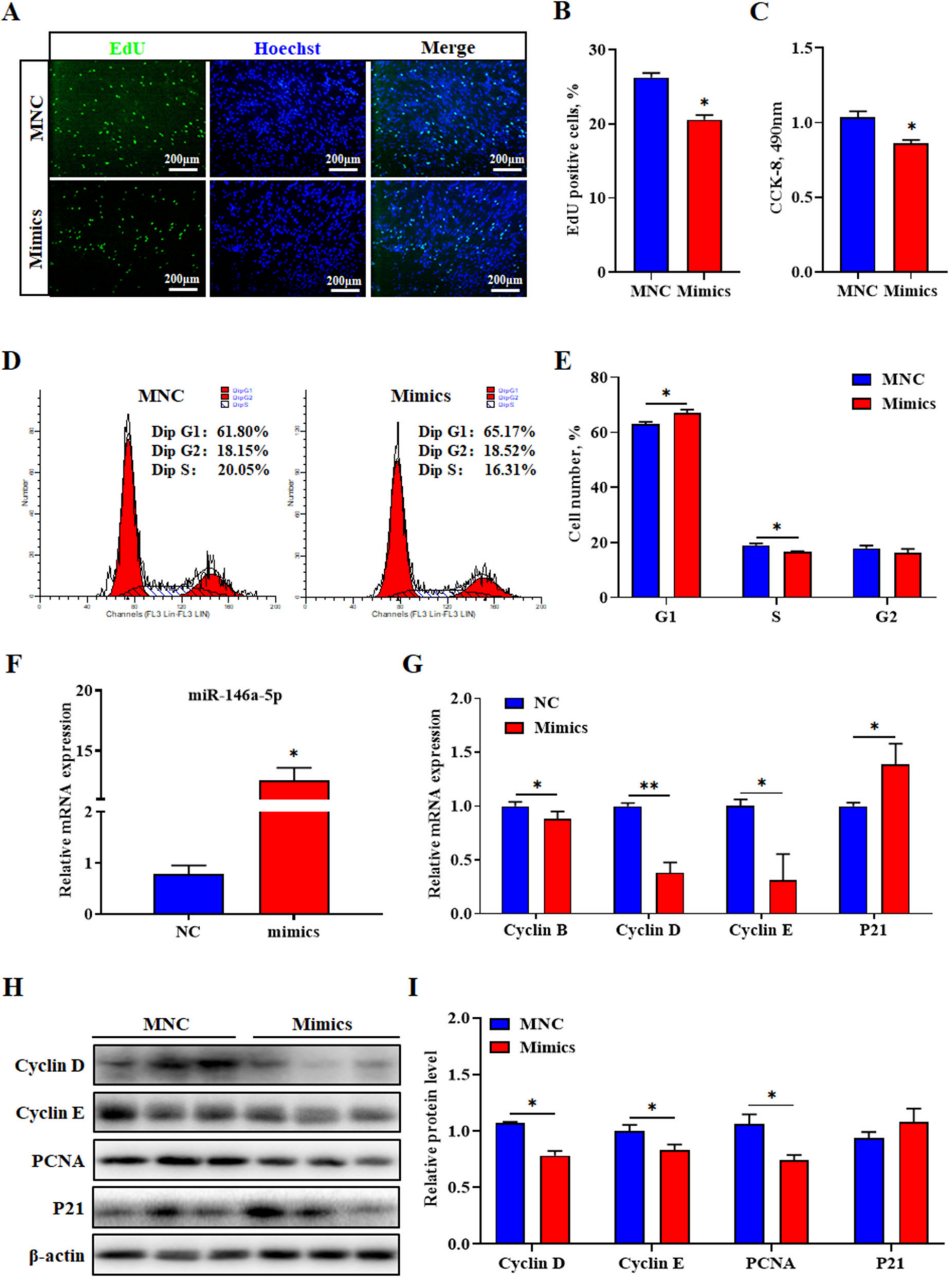
MiR-146a-5p mimics inhibit proliferation of porcine intramuscular preadipocytes. **a** EdU staining assay. Porcine intramuscular preadipocyte in the S-phase were stained with EdU in red, and cell nuclei were dyed with Hoechst in blue. **b** Quantification ratio of EdU-positive cells/total cells. **c** CCK-8 analysis after treatment with miR-146a-5p mimics during porcine intramuscular preadipocyte proliferation. **d** Cell cycle analysis of preadipocyte by flow cytometry. **e** Statistical results of flow cytometry. **f** Overexpression efficiency of miR-146a-5p mimics after transfection for 24 h. **g** RT-qPCR was used to detect the cell cycle genes cyclin B, cyclin D, cyclin E, and p21. **h** Western blot analysis of cyclin E, cyclin D, PCNA and p21 after transfection with miR-146a-5p mimics. **i** Protein quantitative analysis of cyclin E, cyclin D, PCNA and p21. Values are expressed as mean ± SEM (*n* = 3). *, *P* < 0.05; **, *P* < 0.01, versus MNC

To investigate the effect of miR-146a-5p on the proliferation of porcine intramuscular preadipocytes, the miR-146a-5p mimics and mimics negative control (MNC) were transfected into cells. Compared with the MNC group, the positive cells labeled with EDU and the total number of cells in the mimics group was significantly reduced (*P* < 0.05) (Fig. 2a-c). In addition, the number of S-phase cells was significantly lower in the mimics group than in the MNC group (*P* < 0.05), but the number of cells in the G1-phase was significantly higher than that in the MNC group (*P* < 0.05) (Fig. 2d and e). Furthermore, the miR-146a-5p mimics sharply increased the levels of miR-146a-5p (*P* < 0.05), significantly decreased the mRNA levels of cyclin B, cyclin D and cyclin E, whereas it apparently increased the mRNA levels of p21 (*P* < 0.05) (Fig. 2f and g). Similarly, the miR-146a-5p mimics downregulated the protein levels of cyclin D, cyclin E and PCNA (*P* < 0.05), and P21 protein tended to be upregulated, but did not reach statistical significance (Fig. 2h and i).

### MiR-146a-5p inhibitor promotes porcine intramuscular preadipocyte proliferation

**Fig. 3:**
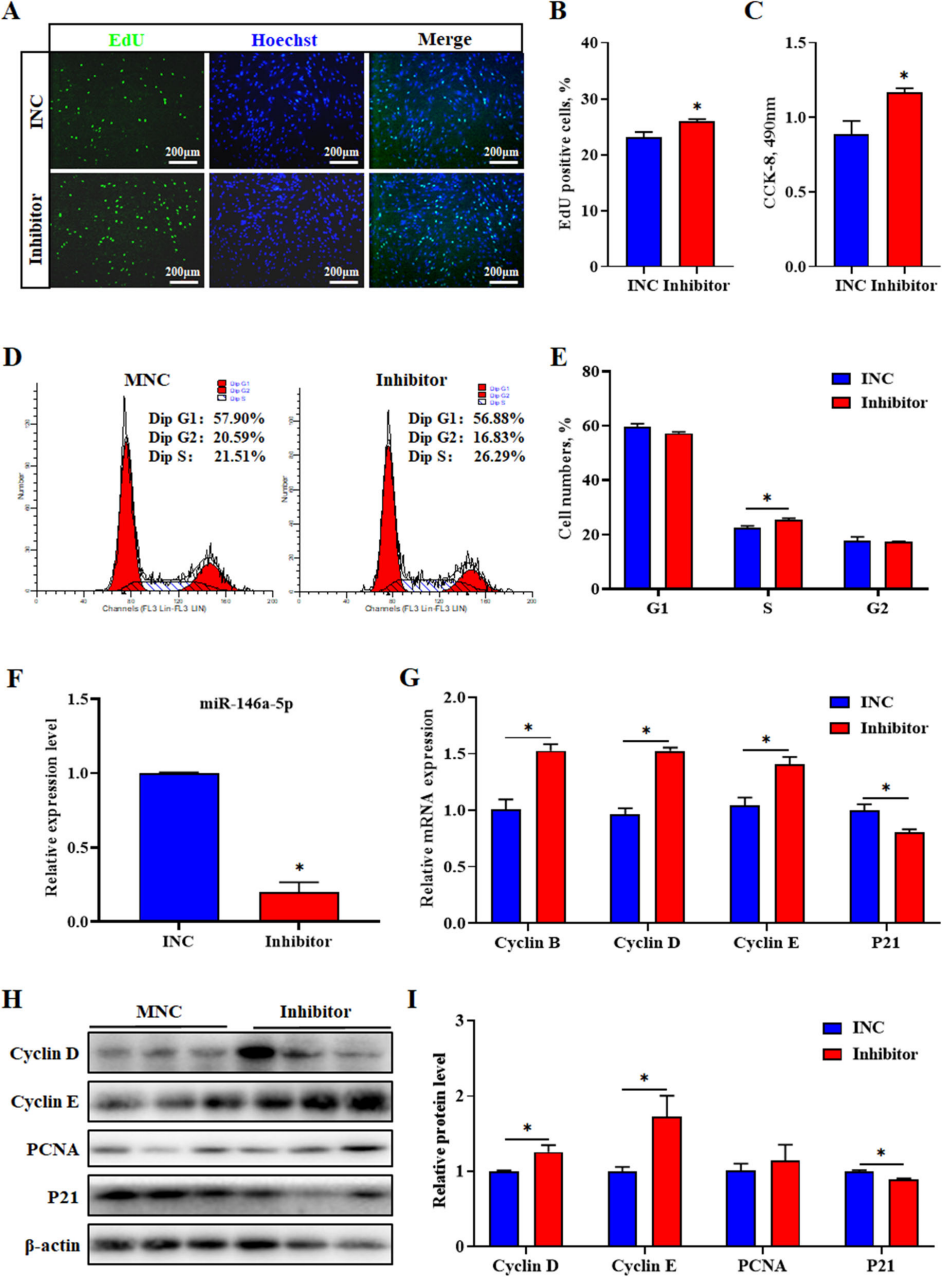
MiR-146a-5p inhibitor promotes porcine intramuscular preadipocyte proliferation. **a** EdU staining assay. Porcine intramuscular preadipocyte in the S-phase were stained with EdU in red, and cell nuclei were dyed with Hoechst in blue. **b** Quantification ratio of EdU-positive cells/total cells. **c** CCK-8 analysis after treatment with miR-146a-5p inhibitor during porcine intramuscular preadipocyte proliferation. **d** Cell cycle analysis of preadipocyte by flow cytometry. **e** Statistical results of flow cytometry. **f** Interference efficiency of miR-146a-5p inhibitor after transfection for 24 h. **g** RT-qPCR was used to detect the cell cycle genes cyclin B, cyclin D, cyclin E, and p21. **h** Western blot analysis of cyclin E, cyclin D, PCNA and p21 after transfection with miR-146a-5p inhibitor. **i** Protein quantitative analysis of cyclin E, cyclin D, PCNA and p21. Values are expressed as the mean ± SEM (*n* = 3). *, *P* < 0.05, versus INC

To validate the role of miR-146a-5p inhibitor on the proliferation of porcine intramuscular preadipocytes, inhibitor negative control (INC) and miR-146a-5p inhibitors were transfected into porcine intramuscular preadipocytes. The results indicated that knockdown of miR-146a-5p significantly increased the number of EdU-positive cells, S-phase cells and total cells (*P* < 0.05) (Fig. 3a-e). The inhibitor effectively decreased the levels of miR-146a-5p (*P* < 0.05) and increased the mRNA levels of cyclin B, cyclin D, and cyclin E, but reduced the level of p21 (*P* < 0.05) (Fig. 3f and g). Meanwhile, the inhibitor upregulated the protein levels of cyclin D and cyclin E, whereas downregulated the protein level of P21 (*P* < 0.05) (Fig. 3h and i).

### MiR-146a-5p targeting *SMAD4* inhibits the proliferation of porcine intramuscular preadipocytes by the TGF-β signaling pathway

**Fig. 4:**
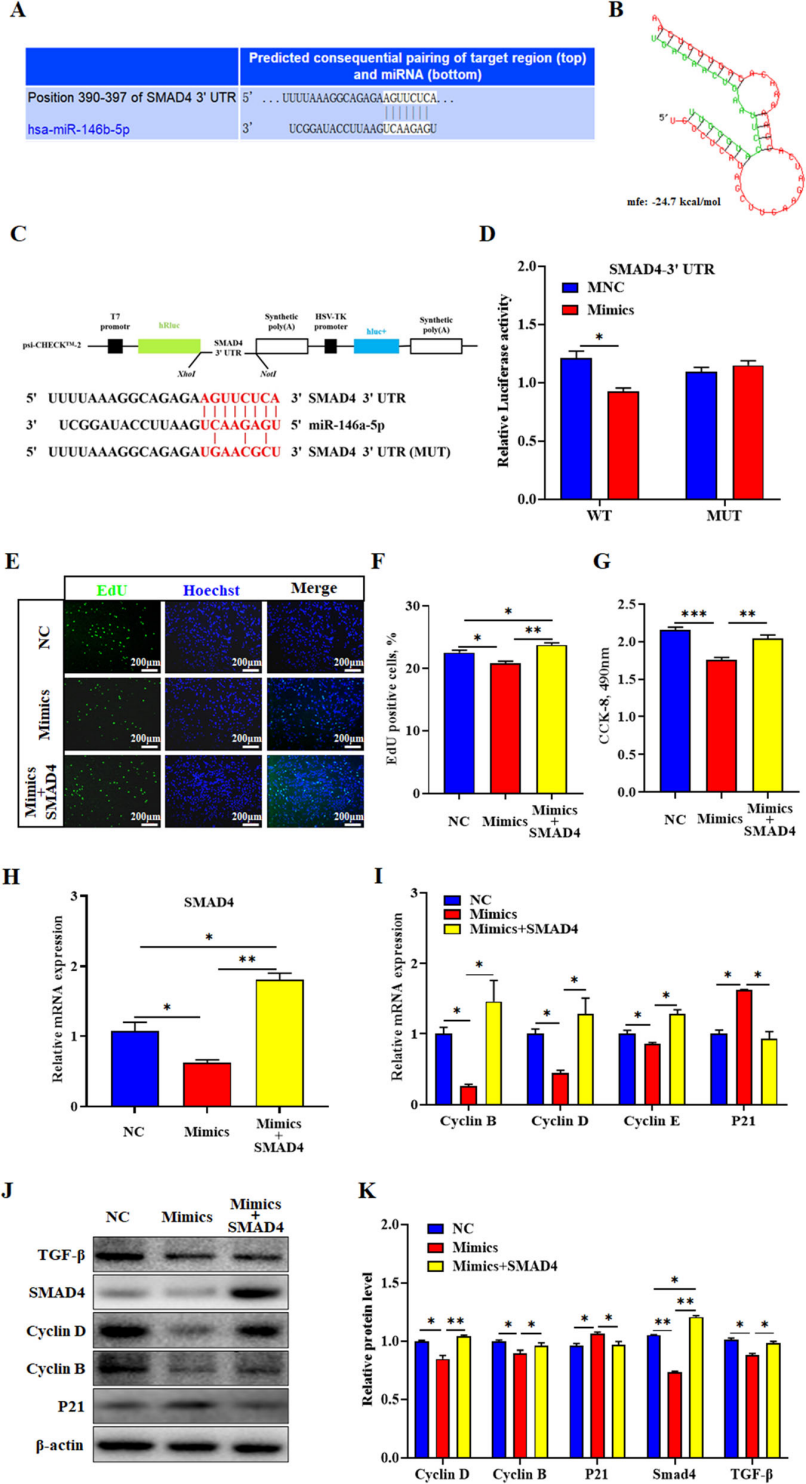
MiR-146a-5p targeting SMAD4 inhibits the proliferation of porcine intramuscular preadipocytes by the TGF-β signaling pathway. **a** *SMAD4* was predicted to be a target of miR-146a-5p by TargetScan software. **b** miR-146a-5p and *SMAD4* 3′ UTR region base complementary pattern diagram. **c** WT and MUT psiCHECK-2.0-*SMAD4* vectors. **d** Relative luciferase activity of *SMAD4* responding to miR-146a-5p mimics. To verify that miR-146a-5p can function by targeting *SMAD4*, we co-treated cells with miR-146a-5p mimics and *SMAD4* overexpression vector (500 ng, 6-well plate). **e** EdU staining assay. **f** Quantification ratio of EdU-positive cells/total cells. **g** CCK-8 analysis. **h** RT-qPCR was used to detect *SMAD4* mRNA expression level. **i** Cell cycle genes cyclin B, cyclin D, cyclin E, and p21 expression level. **j** Western blot analysis of cyclin E, cyclin D, PCNA and p21. **k** Protein quantitative analysis of cyclin E, cyclin D, PCNA and p21. Values are expressed as the mean ± SEM (*n* = 3). *, *P* < 0.05; **, *P* < 0.01; ***, *P* < 0.001, versus NC

To further explore the regulatory mechanism of miR-146a-5p on porcine intramuscular preadipocyte proliferation, we predicted and verified its target genes and signaling pathways. *SMAD4* may be the target gene of miR-146a-5p using TargetScan 7.0 analysis (Fig. 4a and b). The dual-luciferase reporter (DLR) assay results showed that the relative luciferase activity of miR-146a-5p mimics plus *SMAD4* WT vector co-treated group was significantly reduced (*P* < 0.05) (Fig. 4c and d). Next, the rescued experiments were carried out. Compared with the mimics group, the number of EDU-positive cells and the total number of cells in the mimics and *SMAD4* overexpression vector co-treatment group markedly increased (*P* < 0.01), and rescued or even exceeded the NC group level (Fig. 4e-g). Moreover, *SMAD4* overexpression restored the mRNA and protein levels of *SMAD4* and cell cycle-related genes (*P* < 0.05) (Fig. 4h-k). In addition, SMAD4 downstream TGF-β signaling was attenuated by mimics, but was rescued upon *SMAD4* overexpression (*P* < 0.05) (Fig. 4j and k).

### MiR-146a-5p mimics suppress porcine intramuscular preadipocyte differentiation

**Fig. 5:**
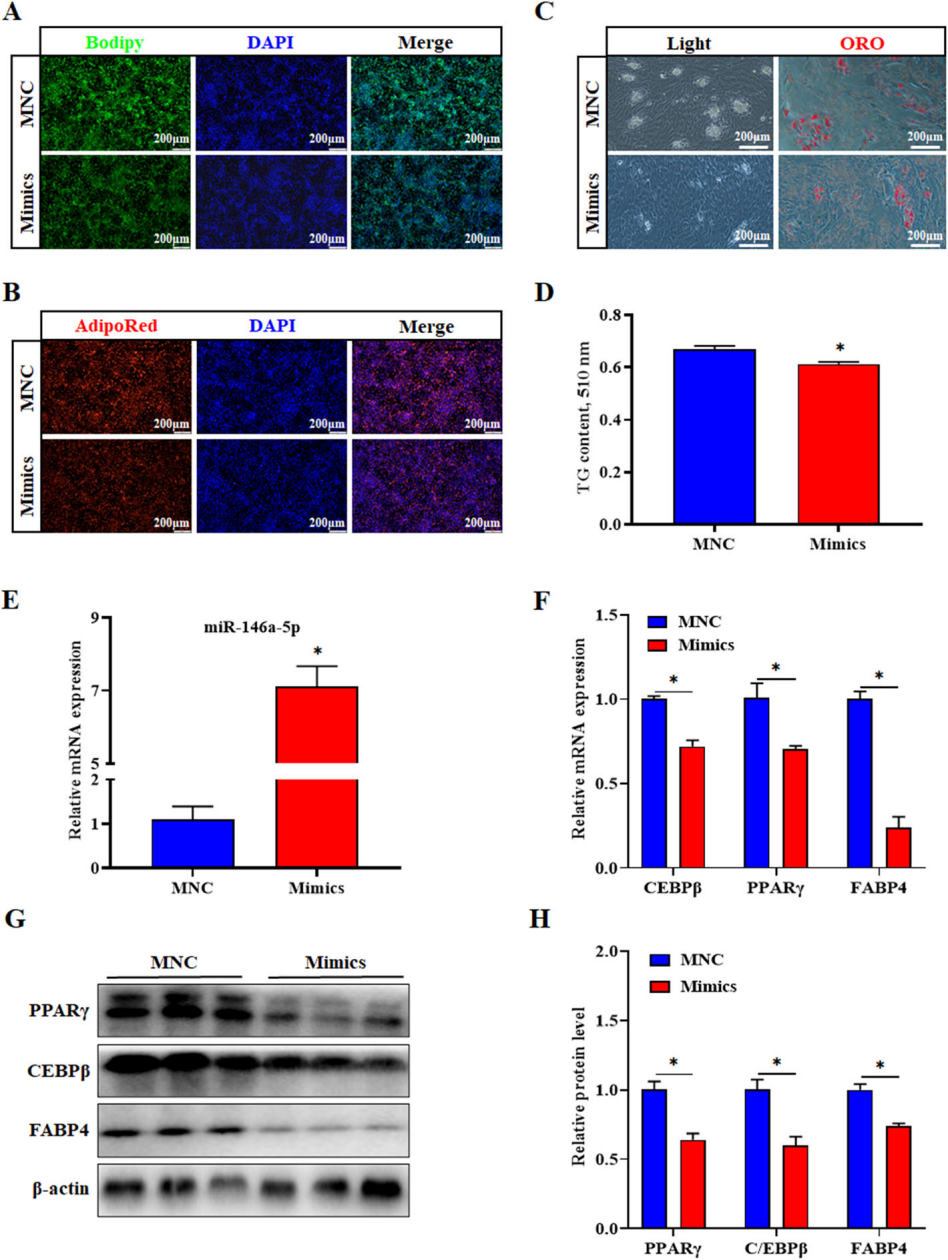
MiR-146a-5p mimics suppresses porcine intramuscular preadipocyte differentiation. Porcine intramuscular preadipocyte were treated with miR-146a-5p mimics to induce differentiation on the 6^th^ day. **a**, **b** Bodipy or AdipoRed staining was performed on lipid droplets. **c** White light field and oil red O stained lipid droplets. **d** After extracting oil red O with isopropanol, the OD value was detected, 510 nm. **e** Overexpression efficiency of miR-146a-5p mimics after transfection for 6 d. **f** RT-qPCR was used to detect the adipogenesis genes *C/EBPβ, PPARγ, and FABP4*. **g** Western blot analysis of C/EBPβ, PPARγ, and FABP4 after transfection with miR-146a-5p mimics. **h** Protein quantitative analysis of **g**. Values are expressed as the mean ± SE (*n* = 3). *, *P* < 0.05, versus MNC

To study the effects of miR-146a-5p mimics on the differentiation of porcine intramuscular preadipocytes, we treated cells with MNC and miR-146a-5p mimics, and then induced adipogenic differentiation for 6 days. Compared with the MNC group, the lipid droplets produced by intramuscular adipocytes apparently decreased in the mimics group, and the triglyceride (TG) content also significantly decreased (*P* < 0.05) (Fig. 5a-d). The expression of miR-146a-5p was significantly increased in differentiated intramuscular adipocytes (*P* < 0.05) (Fig. 5e). However, the mRNA and protein levels of C/EBPβ, PPARγ and FABP4 were both markedly decreased (*P* < 0.05) (Fig. 5f-h).

### MiR-146a-5p inhibitor accelerates porcine intramuscular preadipocyte differentiation

**Fig. 6:**
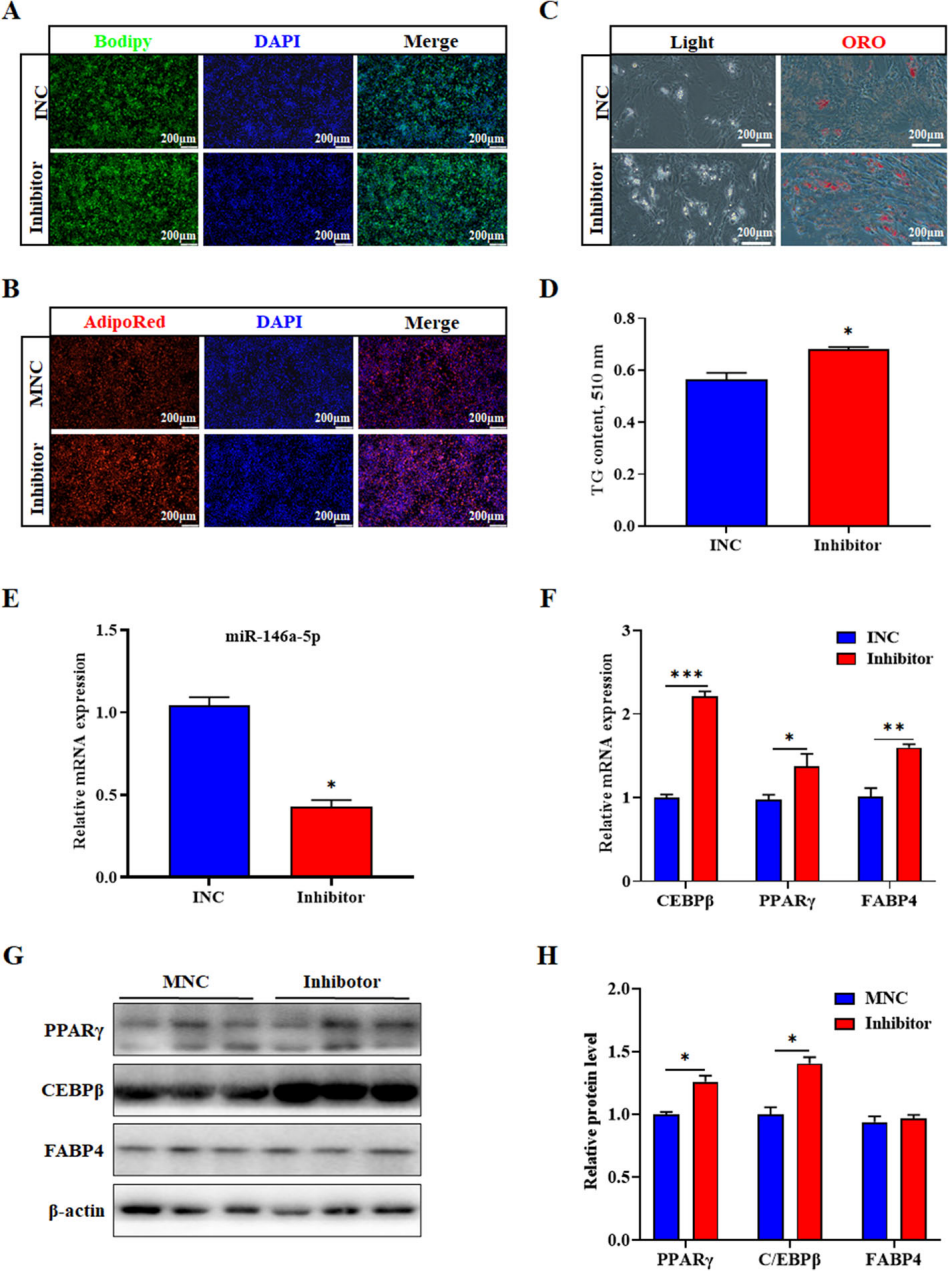
MiR-146a-5p inhibitor accelerates porcine intramuscular preadipocyte differentiation. **a** Porcine intramuscular preadipocyte were treated with miR-146a-5p inhibitor to induce differentiation on the 6th day. **a**, **b** Bodipy or AdipoRed staining was performed on lipid droplets. **c** White light field and oil red O stained lipid droplets. **d** After extracting oil red O with isopropanol, the OD value (510 nm) was detected. **e** Interference efficiency of miR-146a-5p inhibitor after transfection for 6d. **f** RT-qPCR was used to detect the adipogenesis genes *C/EBPβ, PPARγ, and FABP4.* **g** Western blot analysis of C/EBPβ, PPARγ, and FABP4 after transfection with miR-146a-5p inhibitor. **h** Protein quantitative analysis of **g**. Values are expressed as the mean ± SE (*n* = 3). *, *P* < 0.05; **, *P* < 0.01, versus INC

To further validate the role of miR-146a-5p in the differentiation of porcine intramuscular preadipocytes, we carried out the experiments of INC and inhibitor treatment on the cells. Compared with the INC group, the lipid droplets apparently accumulated in intramuscular adipocytes, and the TG content also significantly increased (*P* < 0.05) (Fig. 6a-d). Inhibitor significantly decreased the levels of miR-146a-5p (Fig. 6e), but markedly increased the mRNA levels of *C/EBPβ*, *PPARγ* and *FABP4* (*P* < 0.05) (Fig. 6f). Meanwhile the protein levels of C/EBPβ and PPARγ significantly increased in the treatment group (*P* < 0.05) (Fig. 6g and h).

### MiR-146a-5p targeting *TRAF6* inhibits the differentiation of porcine intramuscular preadipocytes via the AKT/mTORC1 signaling pathway

**Fig. 7:**
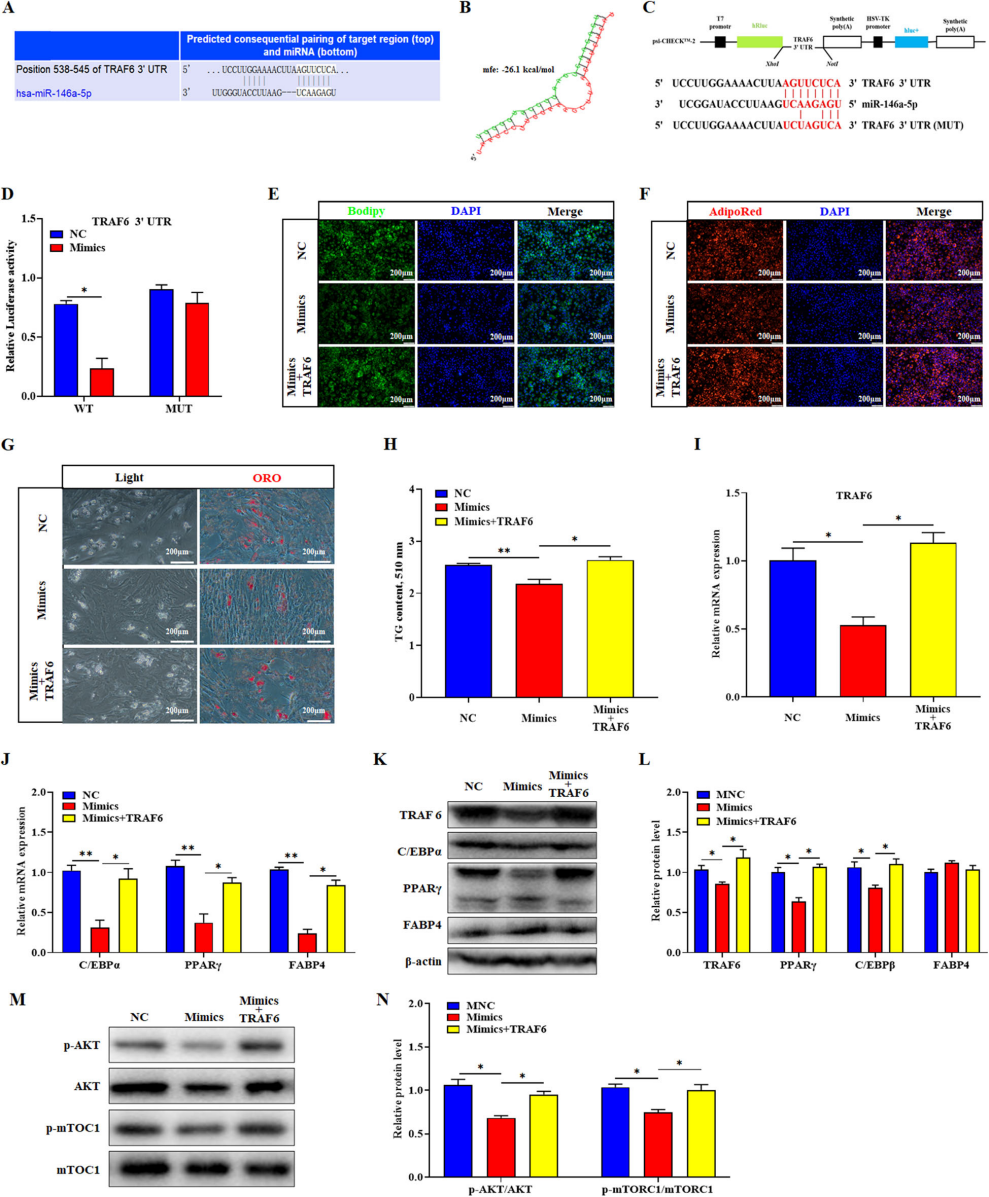
MiR-146a-5p targeting TRAF6 inhibits the differentiation of porcine intramuscular preadipocytes via the AKT/mTORC1 signaling pathway. **a** *TRAF6* was predicted to be a target of miR-146a-5p by TargetScan software. **b** miR-146a-5p and *TRAF6* 3′ UTR region base complementary pattern diagram. **c** WT and MUT psiCHECK-2.0-*TRAF6* vectors. **d** Relative luciferase activity of *TRAF6* responding to miR-146a-5p mimics. To verify that miR-146a-5p can function by targeting *TRAF6*, we co-treated cells with miR-146a-5p mimics and *TRAF6* overexpression vector (500 ng per hole, 6-well plate). **e**, **f** Bodipy or AdipoRed staining was performed on lipid droplets. **g** White light field and oil red O stained lipid droplets. **h** After extracting oil red O with isopropanol, the OD value (510 nm) was detected. **i**, **j** RT-qPCR was used to detect the *TRAF6* and adipogenesis genes *C/EBPβ*, *PPARγ*, and *FABP4*. **k** Western blot analysis of TRAF6, C/EBPβ, PPARγ, and FABP4. **l** Protein quantitative analysis of **k**. **m** Western blot analysis of AKT, p-AKT, mTORC1, and p-mTORC1. **n** Protein quantitative analysis of **m**. Values are expressed as the mean ± SE (*n* = 3). *, *P* < 0.05; **, *P* < 0.01, versus NC

To further investigate the mechanism by which miR-146a-5p regulates the differentiation of intramuscular preadipocytes, we explored its target genes and signaling pathways. The online software predicted that miR-146a-5p could be combined with the *TRAF6* 3′ UTR (Fig. 7a and b). The DLR assay results showed that the relative luciferase activity of miR-146a-5p mimics and *TRAF6* WT vector co-treated group was significantly reduced (*P* < 0.05) (Fig. 7c and d). The rescued experiments were performed. Compared with the mimics group, the lipid droplets and TG content in the *TRAF6* overexpression vector plus mimics co-treated group significantly increased and rescued to the NC group (*P* < 0.05) (Fig. 7e-h). Moreover, *TRAF6* overexpression rescued the mRNA and protein levels of *TRAF6* and adipogenic related genes (*P* < 0.05) (Fig. 7i-l). AKT/mTORC1 as the important downstream signaling pathway of TRAF6, their phosphorylation levels were significantly decreased in mimics group but increased in co-treated group (*P* < 0.01) (Fig. 7m and n).

## Discussion

As a member of the non-coding RNA family, miRNAs have a crucial regulatory role in preadipocyte adipogenesis. Based on bioinformatics analysis of miRNA sequencing data, we found that miR-146a-5p was differentially expressed during SWAT deposition in pigs. Further study showed that the miR-146a-5p sequence was highly conserved, and its function was involved in fat cell proliferation and differentiation by TGF-β and AKT/mTORC1 signal pathways using KEGG and GO analysis. Moreover, miR-146a-5p was highly expressed in porcine white adipose tissue (WAT), and its expression levels first increase and then decrease in proliferated and differentiated porcine intramuscular preadipocytes. Based on the above analysis, we speculated that miR-146a-5p is also implicated in IMF deposition.

It is vital to improve pork quality by controlling the IMF content during pig production. The present study demonstrated that miR-146a-5p plays a crucial role in regulating porcine IMF adipogenesis. miR-146a-5p targets *SMAD4* and inhibits porcine intramuscular preadipocyte proliferation by attenuating TGF-β signaling and also targets *TRAF6* to repress differentiation by weakening AKT/mTORC1 signaling. These findings indicate that miR-146a-5p could be a novel negative regulator of porcine IMF deposition.

IMF deposition depends on the proliferation and differentiation of intramuscular preadipocytes. Our results confirmed that miR-146a-5p inhibited intramuscular preadipocyte proliferation by reducing the number of S-phase cells and downregulating the mRNA and protein levels of cyclin B, cyclin D, cyclin E and PCNA, and upregulating the mRNA and protein levels of p21. Previous studies revealed that miR-146a-5p promotes lung cancer cell proliferation by targeting claudin-12 [33], and overexpression of miR-146 or knockout of its target gene, notch 1, inhibits mouse neural stem cell proliferation in serum-free medium [34]. Therefore, miR-146a-5p differentially modulates the proliferation of different cell types. Generally, genes and miRNAs have opposite effects on cell proliferation and differentiation. Recent studies have shown that miR-664-5p promotes myoblast proliferation and inhibits myoblast differentiation [35], and miR-429 accelerates proliferation of porcine preadipocytes and represses adipogenic differentiation [36]. Interestingly, in this study, miR-146a-5p repressed both the proliferation and differentiation of intramuscular preadipocytes. Studies have indicated that miR-483 inhibits the proliferation and differentiation of bovine myoblasts [37], and miR-342-5p has been found to restrict osteoblast proliferation and differentiation by inhibiting Bmp7 expression [38]. Therefore, our results are reasonable, due to the complexity of intramuscular preadipocyte biological processes that are regulated by miRNAs.

Generally, miRNAs regulate different biological processes in the same cell through different target genes. Therefore, we predicted the target genes of miR-146a-5p that are involved in cell proliferation and adipogenic differentiation, respectively. During the proliferation phase, we predicted that *SMAD4* was the target gene of miR-146a-5p. Recent studies have shown that miR-145-5p inhibits ovarian epithelial cancer cell proliferation by targeting *SMAD4* [39], and miR-663a overexpression suppresses hepatic stellate cell proliferation by downregulating *SMAD4* levels [40]. Based on the above results, *SMAD4* functions mostly as a positive regulator of cell proliferation. Moreover, the *SMAD4* and TGF-β signaling pathways play important roles in miRNA regulation of cell proliferation [41, 42]. Notably, miR-183 promotes preadipocyte adipogenesis by suppressing *SMAD4* expression in goats [43], and myostatin/SMAD4 signaling inhibits 3T3-L1 cell differentiation [44]. These findings demonstrate that the effects of SMAD4 on adipocyte differentiation vary in different cell types. However, in our study, *SMAD4* was not identified as a target gene of miR-146a-5p during the differentiation phase of porcine intramuscular preadipocytes (Fig. S2A and B). Here, we revealed that miR-146a-5p is a novel miRNA that targets *SMAD4* to repress porcine intramuscular preadipocyte proliferation via the TGF-β signaling pathway.

Furthermore, we confirmed through TargetScan 7.0 analysis, luciferase activity assay, and rescue experiments that during porcine intramuscular preadipocyte adipogenic differentiation, the target gene of miR-146a-5p is *TRAF6*. As expected, miR-146a-5p targeted *TRAF6* during cell differentiation, but not during proliferation (Fig. S2C and D). TRAF6 is a signal transduction factor that connects cell surface receptors with intracellular signal proteins. In addition to the inflammatory immune response, *TRAF6* also regulates cell differentiation and survival [45]. Previous studies have shown that inhibiting the CD40-TRAF6 interaction induces obesity by improving glucose tolerance and reducing the accumulation of immune cells into adipose tissue [46]. Moreover, green tea extracts reduced the adipose tissue weight of obese mice by reducing *TRAF6* expression [47]. Interestingly, a significant reduction in wet weight and adipocyte hypertrophy was observe in epididymal WAT of adipocyte-specific TRAF6-KO mice on a high fat diet (HFD), which suggests TRAF6 inhibition in adipocytes could relieve the obesity induced by a HFD [19]. Therefore, inhibiting TRAF6 expression reduce the accumulation of fat to relieve obesity. However, it is not clear whether inhibiting TRAF6 to prevent obesity is caused by affecting adipocyte differentiation. Notably, AKT/mTORC1 signaling, is downstream of TRAF6, is indispensable in the preadipocyte differentiation process [21, 48, 49]. In addition, the process of adipogenesis is often accompanied by cell inflammation. In our study, when the adipogenesis of porcine intramuscular preadipocyte was inhibited, the inflammatory factor NF-κB and its phosphorylation levels were also downregulated, and the adipogenic ability and inflammation were restored after the addition of the TRAF6 overexpression vector (Fig. S3A and B). In general, we found that miR-146a-5p targets *TRAF6* to inhibit porcine intramuscular preadipocyte differentiation through regulating the AKT/mTORC1 signaling pathway.

## Conclusions

**Fig. 8:**
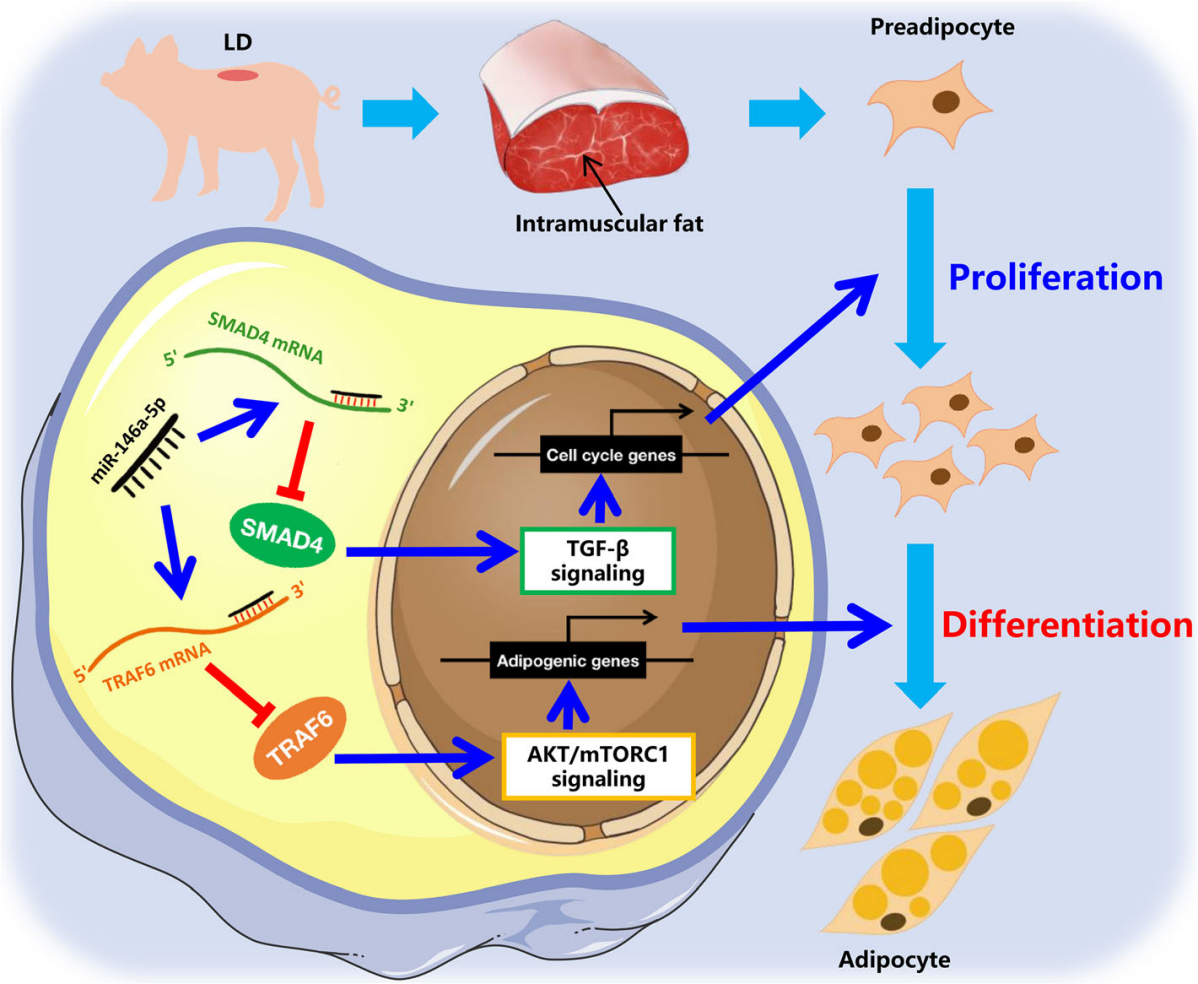
A model depicting the role of miR-146a-5p in regulating porcine IMF adipogenesis. The intramuscular preadipocytes are derived from the pig’s longest dorsal muscle. On the one hand, miR-146a-5p targets *SMAD4* mRNA, inhibits the formation of signal transduction factors, inhibits TGF-β signal transmission into the nucleus, and inhibits cell proliferation by inhibiting cell cycle related genes. On the other hand, miR-146a-5p directly targets *TRAF6* mRNA to inhibit its translation and inhibits adipogenesis gene expression through the AKT/mTORC1 signaling pathway, thereby inhibiting adipogenesis

In conclusion, miR-146a-5p targets *SMAD4* to inhibit porcine intramuscular preadipocyte proliferation through the TGF-β signaling pathway, and miR-146a-5p also targets *TRAF6* to repress adipogenic differentiation via the AKT/mTORC1 signaling pathway (Fig. 8). These findings provide a novel miRNA biomarker for modulating IMF content to enhance pork quality and help us to better understand the role and regulatory mechanism of miRNAs in IMF adipogenesis.

## Supplementary Information

**Additional file 1: Figure S1.** Porcine intramuscular preadipocytes of different densities. A, white light field of newly seeded porcine intramuscular preadipocyte. B, white light field of porcine intramuscular preadipocyte which the density reached 50–60%. C, white light field of porcine intramuscular preadipocyte which the density reached 90–100%.

**Additional file 2: Figure S2.** The expression of TRAF6 and SMAD4 during the proliferation and differentiation of porcine intramuscular preadipocytes. A, western blot analysis of SMAD4 in differentiated porcine intramuscular adipocytes which transfected with miR-146a-5p mimics. B, protein quantitative analysis of A. C, western blot analysis of TRAF6 in proliferation porcine intramuscular adipocytes which transfected with miR-146a-5p mimics. D, protein quantitative analysis of C. Values are expressed as mean ± SEM (*n* = 3).

**Additional file 3: Figure S3.** The protein levels of NF-κB and p-NF-κB. A, western blot analysis of NF-κB and p-NF-κB in differentiated porcine intramuscular adipocytes. B, protein quantitative analysis of A. Values are expressed as mean ± SEM (*n* = 3). *, *P* < 0.05; **, *P* < 0.01, versus MNC.

## Data Availability

All data generated or analyzed during this study are available from the corresponding author by request.

## Abbreviations

IMF: Intramuscular fat
miRNAs: MicroRNAs
SMAD4: SMAD family member 4
TRAF6: TNF receptor associated factor 6
WAT: White adipose tissue
CDKs: Cyclin-dependent kinases
CKIs: CDK inhibitors
PPARγ: Peroxisome proliferator-activated receptor γ
C/EBP: CCAAT/enhancer binding protein
FABP4: Fatty acid binding protein 4
LPL: Lipoprotein lipase
LD: Longissimus dorsi
SWAT: Subcutaneous white adipose tissue
PBS: Phosphate buffer saline
RT-qPCR: Real-time quantitative PCR
MNC: Mimics negative control
INC: Inhibitor negative control
DLR: Dual-luciferase reporter
TG: Triglycerides
PI: Propidium iodide
