# Supplementary figures and images for: MiR-146a-5p targeting SMAD4 and TRAF6 inhibits adipogenensis through TGF-β and AKT/mTORC1 signal pathways in porcine intramuscular preadipocytes
Source: J Anim Sci Biotechnol. 2021 Feb 3;12:12. doi: 10.1186/s40104-020-00525-3 (PMC7856799; doi:10.1186/s40104-020-00525-3)

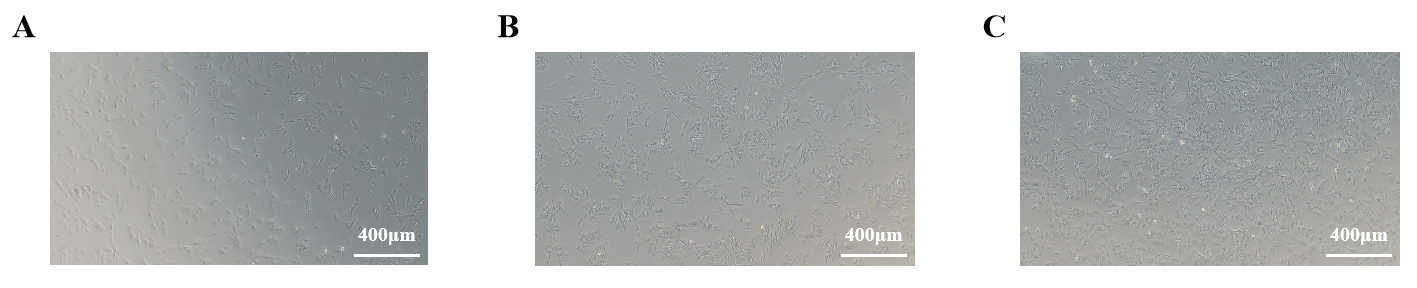

Supplement: Supplementary file 1 — Additional file 1: Figure S1. Porcine intramuscular preadipocytes of different densities. A, white light field of newly seeded porcine intramuscular preadipocyte. B, white light field of porcine intramuscular preadipocyte which the density reached 50–60%. C, white light field of porcine intramuscular preadipocyte which the density reached 90–100%. [file 40104_2020_525_MOESM1_ESM.tif]

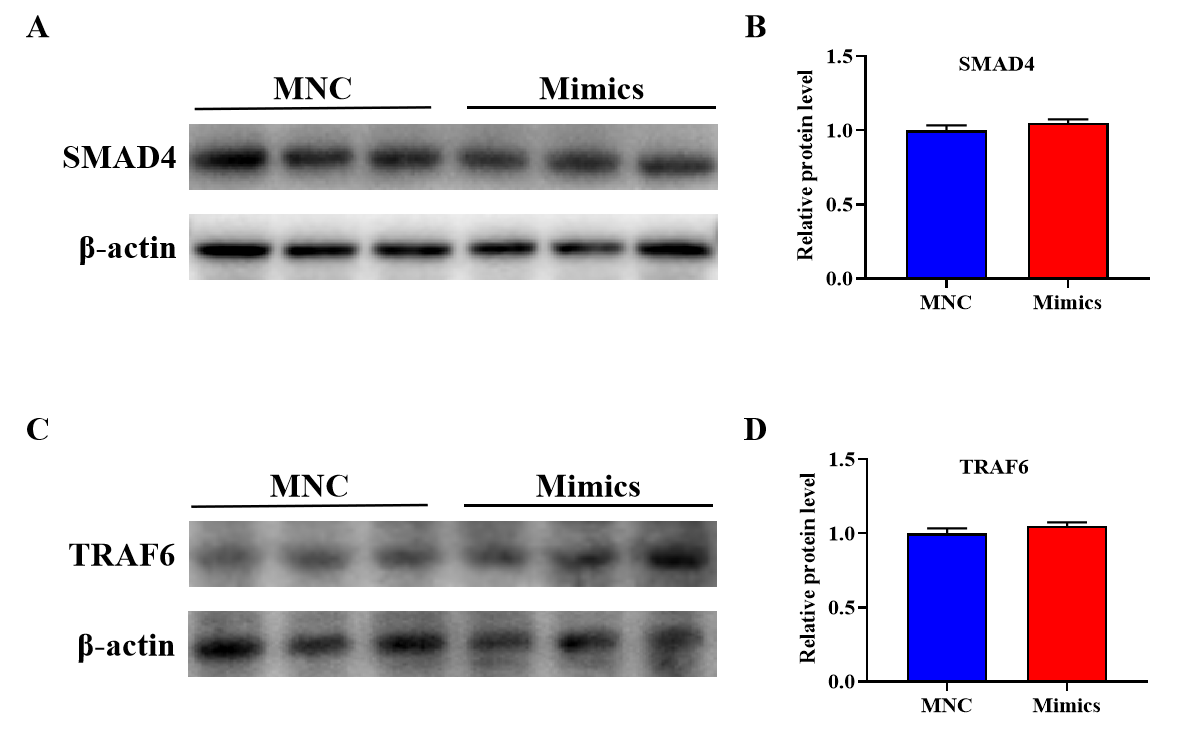

Supplement: Supplementary file 2 — Additional file 2: Figure S2. The expression of TRAF6 and SMAD4 during the proliferation and differentiation of porcine intramuscular preadipocytes. A, western blot analysis of SMAD4 in differentiated porcine intramuscular adipocytes which transfected with miR-146a-5p mimics. B, protein quantitative analysis of A. C, western blot analysis of TRAF6 in proliferation porcine intramuscular adipocytes which transfected with miR-146a-5p mimics. D, protein quantitative analysis of C. Values are expressed as mean ± SEM (n = 3). [file 40104_2020_525_MOESM2_ESM.tif]

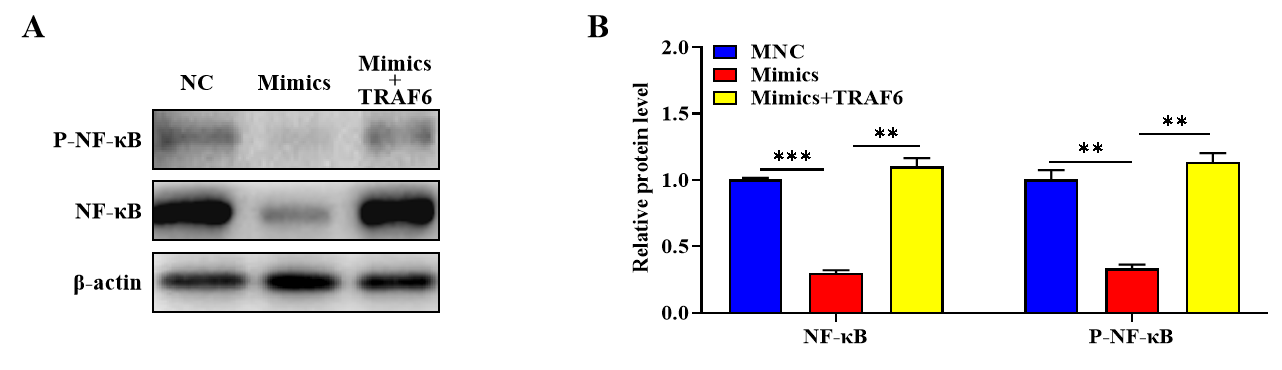

Supplement: Supplementary file 3 — Additional file 3: Figure S3. The protein levels of NF-κB and p-NF-κB. A, western blot analysis of NF-κB and p-NF-κB in differentiated porcine intramuscular adipocytes. B, protein quantitative analysis of A. Values are expressed as mean ± SEM (n = 3). *, P < 0.05; **, P < 0.01, versus MNC. [file 40104_2020_525_MOESM3_ESM.tif]
